# Nationwide estimates of all-cause and cause-specific mortality among individuals with gender dysphoria in Sweden, 2001–2023: a register-based cohort study

**DOI:** 10.1016/j.eclinm.2026.104088

**Published:** 2026-07-31

**Authors:** Kristen D. Clark, Richard A. White, Sarah S. Jackson, Alkistis Skalkidou, Thomas Frisell, Fotios C. Papadopoulos

**Affiliations:** a Department of Medical Sciences, Clinical Psychiatry, Uppsala University, Uppsala, Sweden; b Department of Women's and Children's Health, Uppsala University, Uppsala, Sweden; c Norwegian Institute of Public Health, Oslo, Norway; d Blizzard Institute, Queen Mary University of London, London, UK; e Division of Clinical Epidemiology, Department of Medicine Solna, Karolinska Institute, Stockholm, Sweden

**Keywords:** Transgender, Gender dysphoria, Mortality, Register-based study, Health disparities, Cohort study

## Abstract

**Background:**

Transgender and gender-diverse (TGD) people experience elevated mortality risk, yet population-based estimates of cause-specific mortality remain limited. Prior studies have focused on narrow subgroups or outdated cohorts, leaving gaps in understanding current mortality disparities. Therefore, the purpose of this study is to quantify all-cause and cause-specific mortality among all TGD adults with a diagnosis of gender dysphoria in Sweden.

**Methods:**

This cohort study provides national cause-specific mortality estimates among TGD adults in Sweden using registry data (2001–2023). TGD people were identified using ICD-10 codes for “gender dysphoria” and matched to approximately 10 cisgender controls of each sex by age and county. Age-standardized mortality rates, absolute mortality risk differences, mortality rate ratios (MRR), and years of potential life lost (YPLL) were calculated overall and stratified by sex assigned at birth.

**Findings:**

A total of 9038 TGD people were identified, among them, 211 deaths were observed. All-cause mortality risk was almost twice that of the cisgender comparison group (MRR = 1.9, 95% confidence interval [CI] 1.7, 2.2). Stratified analyses showed distinct mortality patterns for transfeminine (assigned male sex at birth) and transmasculine (assigned female sex at birth) people, with higher risk from ill-defined diseases among transfeminine people (cisgender women as reference: MRR = 4.9, 95% CI 2.3, 10.3; cisgender men: MRR = 5.5, 95% CI 2.6, 11.4) and transmasculine people from neoplasms (cisgender women: MRR = 2.4, 95% CI 1.4, 4.1; cisgender men: MRR = 1.8, 95% CI 1.1, 3.1). These disparities resulted in a mean of 6.3 YPLL for transfeminine people compared to cisgender men and 9.0 YPLL for transmasculine people compared to cisgender women.

**Interpretation:**

These findings highlight persistent health inequities and underscore the need for targeted public health interventions and an evaluation of data collection processes to improve health monitoring and support TGD populations in Sweden.

**Funding:**

The present study was funded by a Starting Grant (2023-01397) and a project grant (2021-01968) from the Swedish Research Council for Health, Working Life and Welfare (FORTE).

**Research in context**

**Evidence before this study**

We searched PubMed and Google Scholar for studies published between January 2000 and September 2025 using terms including [“transgender,” OR “gender dysphoria,”] AND “mortality.” Articles were restricted to English-language and peer-reviewed studies. Previous research has consistently shown elevated mortality risk, overall and specifically due to suicide and external causes, among transgender and gender-diverse (TGD) people (i.e., those whose experienced gender is discordant from their sex assigned at birth) compared to cisgender populations, or people who do not experience a discordance between their gender and sex assigned at birth. In Sweden, prior research examined mortality only among people who had legally changed their gender between 1973 and 2003, limiting generalizability. Studies from the United Kingdom, Netherlands, and Denmark have reported elevated mortality risks but varied in their inclusion criteria, adjustment for confounders, and outcome definitions. Few studies have examined cause-specific mortality across a broad TGD population using national registry data, and even fewer report both absolute and relative measures of mortality inequality.

**Added value of this study**

This study provides the most current and comprehensive registry-based national estimates of all-cause and cause-specific mortality among TGD people in Sweden who have had contact with the gender-affirming healthcare system. It includes over 9000 TGD adults matched to cisgender controls of both sexes, allowing for robust comparisons. We show both relative and absolute measures of mortality risk, including age-standardized mortality rates, mortality risk ratios, and years of life lost. Stratified analyses highlight distinct mortality patterns for transfeminine (assigned male sex at birth) and transmasculine people (assigned female sex at birth). Notably, we found elevated mortality risk from external causes, ill-defined diseases, and neuropsychiatric conditions among transfeminine people and infectious/parasitic and respiratory diseases among transmasculine people, while some causes varied by reference group. These findings expand upon disparities identified in Swedish and international studies, suggesting systemic gaps in healthcare access and potential biases in death certification practices.

**Implications of all the available evidence**

These findings underscore persistent health inequities faced by TGD people across multiple mortality causes. This emphasizes the need for targeted public health interventions, improved death certification processes, and inclusive healthcare policies. The high mortality from ill-defined diseases, particularly among transfeminine people, may reflect systemic barriers in healthcare access or documentation practices. Differences in mortality patterns by sex assigned at birth suggest that tailored approaches are needed to address the distinct health risks faced by transfeminine and transmasculine populations. Future research should explore the underlying mechanisms driving these disparities, including stigma as a form of stress and barriers to healthcare access, as well as biological, behavioral, and administrative factors, and establish causal pathways linking clinical treatment to mortality outcomes.

## Introduction

In recent years, the number of people seeking treatment for gender dysphoria, a clinical term describing the distress that arises when a person's gender identity does not align with their sex assigned at birth, has risen markedly. Transgender and gender diverse (TGD) people have long been observed to have poor mental and physical health outcomes, which have been associated with numerous types of stigma (minority stress).^1^ The World Health Organization has recognized the need for targeted efforts to improve the health and healthcare access of TGD people.^2^ Among known health disparities, TGD people have widely been observed to have higher mortality rates when compared to cisgender (those whose gender is aligned solely with their sex assigned at birth) people in numerous studies.^3^

Previous research on mortality risk among TGD people has shown increased risk across numerous outcomes when compared to cisgender people.^3^ A United States (US) study of privately insured cohorts (2011–2019) reported almost a two-fold greater all-cause mortality risk for TGD people when compared to cisgender controls.^4^ Similarly, a study in the United Kingdom (UK) found between 34% and 75% greater all-cause mortality risk for TGD people.^5^ Suicide consistently emerges as a leading cause-specific contributor to excess mortality.^6^, ^7^, ^8^, ^9^ While the consensus indicates elevated mortality, one notable exception is a US Veterans Administration claims analysis (1999–2016), which, despite confirming increased suicide risk, reported a 10% lower all-cause mortality risk.^8^ This finding deviates from the broader literature on TGD mortality outcomes.

Prior studies are limited by heterogeneous inclusion criteria, most of which only examined TGD people receiving gender-affirming treatment or deaths specifically from suicide.^7^, ^8^, ^9^, ^10^, ^11^, ^12^ Within Sweden, mortality risk has only been studied among TGD people who had obtained a legal gender change between 1973 until 2003.^6^ Until 2013, legal gender change required TGD people to be unmarried and undergo sterilization, which means that this subset is not representative of the total TGD population as some may not opt to receive gender-affirming treatments (including hormone therapy and surgery).^13^^,^^14^ Recent years have seen an increase in TGD people seeking evaluation and gender-affirming treatment, as well as a rise in psychological comorbidities and in the proportion assigned female sex at birth.^13^^,^^15^ Structural barriers and restrictions to social recognition of TGD identities have also shifted, such as the repeal of Swedish sterilization requirements for legal gender recognition in 2013, which expands the choices for gender-affirming treatment offered to TGD people.^16^ Similar demographic and legislative shifts have occurred internationally.^17^, ^18^, ^19^ Consequently, existing mortality estimates may not reflect today's more diverse TGD population. Therefore, using population-based Swedish registers, we aim to quantify all-cause and cause-specific mortality among all TGD adults with a diagnosis of gender dysphoria in Sweden.

## Methods

### Study design and setting

We conducted a cohort study using data from Swedish registers (Total Population Register, National Patient Register, and National Cause of Death Register) between 2001 and 2023 to address the study's aim. The National Patient Register provides data on diagnosis codes related to inpatient and outpatient healthcare encounters with International Classification of Diseases version 10 (ICD-10)^20^ and NOMESCO Classification of Surgical Procedures codes.^21^

### Participants

Data provided by the National Board of Health and Welfare included all people in Sweden who had ICD-10 codes for gender dysphoria (i.e., F64.0, F64.8, or F64.9) from the age of 10 or older. A group of controls (approximately 10 individuals of the same sex assigned at birth and approximately 10 of the opposite sex for each case) was provided by Statistics Sweden, matched by age and county. Controls that were also cases were subsequently excluded.

### Variables

Age was calculated based on study-related events (i.e., first gender dysphoria diagnosis, death, end of data collection period). Causes of death and the accompanying dates were determined based on the ICD-10 codes in two phases (Supplement 1). All causes of death variables (underlying and contributing causes) were first searched for codes related to external causes. This is because the circumstances around external causes of death can be multifactorial and result in inconsistencies in the attribution of underlying causes.^22^, ^23^, ^24^ The remaining deaths were categorized based on the ICD-10 codes designated as the underlying cause.^25^ These codes were grouped based on the ICD-10, version 2019 classifications.^21^

### Data sources and management

A 10-digit National Registration Number serves as a unique personal identifier assigned to all Swedish residents. This personal identifier is used in the linkage of records between registers to specific people. To protect participant privacy, National Registration Numbers are replaced with new identifiers before the data is provided to researchers and the key is then destroyed.

### Statistical methods

Descriptive statistics were used to describe the characteristics of the sample (TGD people and matched cisgender people) and deaths (all-cause and cause-specific). Categorical variables were presented as counts and percentages. Continuous variables were assessed for normality and presented as mean (standard deviation). Given the matched design of this study, formal statistical testing of baseline characteristics was not performed, as standard tests assume independence between groups. The mortality rate was age-standardized based on 2023 Swedish population statistics^26^ and reported per 10,000 person-years, as was the absolute mortality rate difference (ARD). The mortality rate ratios (MRR) were calculated as the ratio of age-standardized mortality rates between TGD people and matched cisgender controls. Confidence intervals were derived using the Dobson method, which assumes Poisson-distributed event counts. Analyses were stratified by sex assigned at birth, where those assigned female sex at birth are referred to as transmasculine and those assigned male sex at birth are referred to as transfeminine to more closely align with their gender identity. Stratified TGD samples were then compared to matched sex controls and opposite sex controls. This is because the hormone milieu and body composition of TGD people can change over time, making same sex assigned at birth comparisons unclear in their interpretation. This approach also helps to inform whether disparities are unique to TGD status or reflect broader sex and gender-related patterns.^5^^,^^27^ The observed years of potential life (YPLL) lost were calculated with a selected cut-off of 83 years.^26^ People who died at age ≥83 years contributed 0 YPLL. The total YPLL was summed and divided by the number of deaths within each stratum, yielding the mean YPLL per decedent. The difference in mean YPLL for TGD people against matched cisgender people was then calculated. The projected YPLL for the total sample was obtained by multiplying the number of TGD people by the difference in mean YPLL to obtain a population-level estimation of premature mortality burden.^28^ R-statistical software environment was used for data management and analysis (R version 4.3.3).^29^

### Ethical approval

This study was approved and informed consent was waived by the Central Ethical Review Board in Stockholm (Dnr. Ö30-2016). All methods were performed per the relevant guidelines and regulations.

### Role of the funding source

The funders had no role in considering the study design or in the collection, analysis, interpretation of data, writing of the report, or decision to submit the article for publication.

## Results

After excluding controls that were also cases (*n* = 11) and people with gender dysphoria-related ICD codes prior to version 10 (*n* = 10), a total of 9038 TGD people were included in the analysis (Table 1). During the study period, a total of 211 deaths were documented among TGD people and 2187 among cisgender people (*n* = 179,816). The TGD cohort was predominantly born in Sweden (83.7%), although the proportion of people born outside the Nordic countries was slightly higher among transfeminine participants (18.5%) compared to matched cisgender women (19.5%) and men (20.4%) control groups. The average age at cohort entry was 24.5 (SD = 11.4) for TGD people and 24.6 (SD = 11.4) for cisgender people. Notably, the transfeminine subgroup had a higher mean age at entry (28.7 years) compared to the transmasculine subgroup (24.0 years), reflecting the demographic composition of the Swedish gender dysphoria diagnosis cohort during the study period. The total person-years accumulated were 70,900 for TGD people and 1,490,000 for cisgender people, with a mean of 7.0 years of follow-up for both groups. The all-cause MRR was almost double for TGD people (ASMR = 183.2 per 10,000 person-years, 95% CI 159.3, 209.7) compared to cisgender people (MR = 96.3, 95% CI 92.3, 100.4; MRR: 1.9, 95% CI: 1.7, 2.2).

**Table 1:** Characteristics of transgender and gender diverse (TGD) people and matched cisgender people in Swedish register data between 2001 and 2023, total sample and stratified by sex assigned at birth.

Characteristic	Total sample cohort	Total sample controls	Transfeminine people and matched cisgender women and men			Transmasculine people and matched cisgender women and men		
	TGD	Cisgender	Transfeminine people	Cisgender women	Cisgender men	Transmasculine people	Cisgender women	Cisgender men
	*n* = 9038	*n* = 179,816	*n* = 4181	*n* = 41,654	*n* = 41,659	*n* = 4857	*n* = 48,259	*n* = 48,244
Country of birth (n, %)								
Sweden	8459 (83.7)	168,473 (79.5)	3339 (79.9)	32,951 (79.1)	32,644 (78.4)	4126 (84.9)	38,513 (79.8)	37,941 (78.6)
Another Nordic Country	158 (1.6)	2354 (1.1)	67 (1.6)	588 (1.4)	506 (1.2)	82 (1.7)	592 (1.2)	410 (0.8)
Outside of Nordics	1487 (14.7)	411,146 (19.4)	774 (18.5)	8111 (19.5)	8504 (20.4)	647 (13.3)	9153 (19.0)	9887 (20.5)
Missing			1 (0.0)	4 (0.0)	5 (0.0)	2 (0.0)	1 (0.0)	6 (0.0)
Age at entry, mean [M] (Standard Deviation [SD])	24.5 (11.4)	24.6 (11.4)	28.7 (12.2)	28.8 (12.2)	28.7 (12.2)	24.0 (8.9)	24.1 (8.9)	24.1 (8.9)
Age at exit, M (SD)	30.0 (13.4)	30.2 (13.6)	34.6 (14.4)	34.8 (14.6)	34.7 (14.5)	29.3 (11.1)	29.4 (11.2)	29.4 (11.1)
Person-years, M (SD)	7.0 (5.0)	7.0 (5.1)	7.3 (5.5)	7.4 (5.6)	7.4 (5.6)	6.7 (4.9)	6.7 (4.9)	6.7 (4.9)
Total person-years	70,900	1,490,000	30,664	309,892	308,035	32,305	323,034	322,643
Deaths during the study period	211	2187	138	585	831	73	312	459
Years of life lost compared to cisgender women, M^a^	–	–	8.8	–	–	9.0	–	–
Projected population-level of years of life lost estimate compared to cisgender women^a^	–	–	36,792.8	–	–	43,713.0	–	–
Years of life lost compared to cisgender men, M^a^	–	–	6.3	–	–	2.7	–	–
Projected population-level of years of life lost estimate compared to cisgender men^a^	–	–	26,340.3	–	–	13,113.9	–	–

a Years of life lost are based on the difference in age of death between case and control groups.

**Fig. 1:**
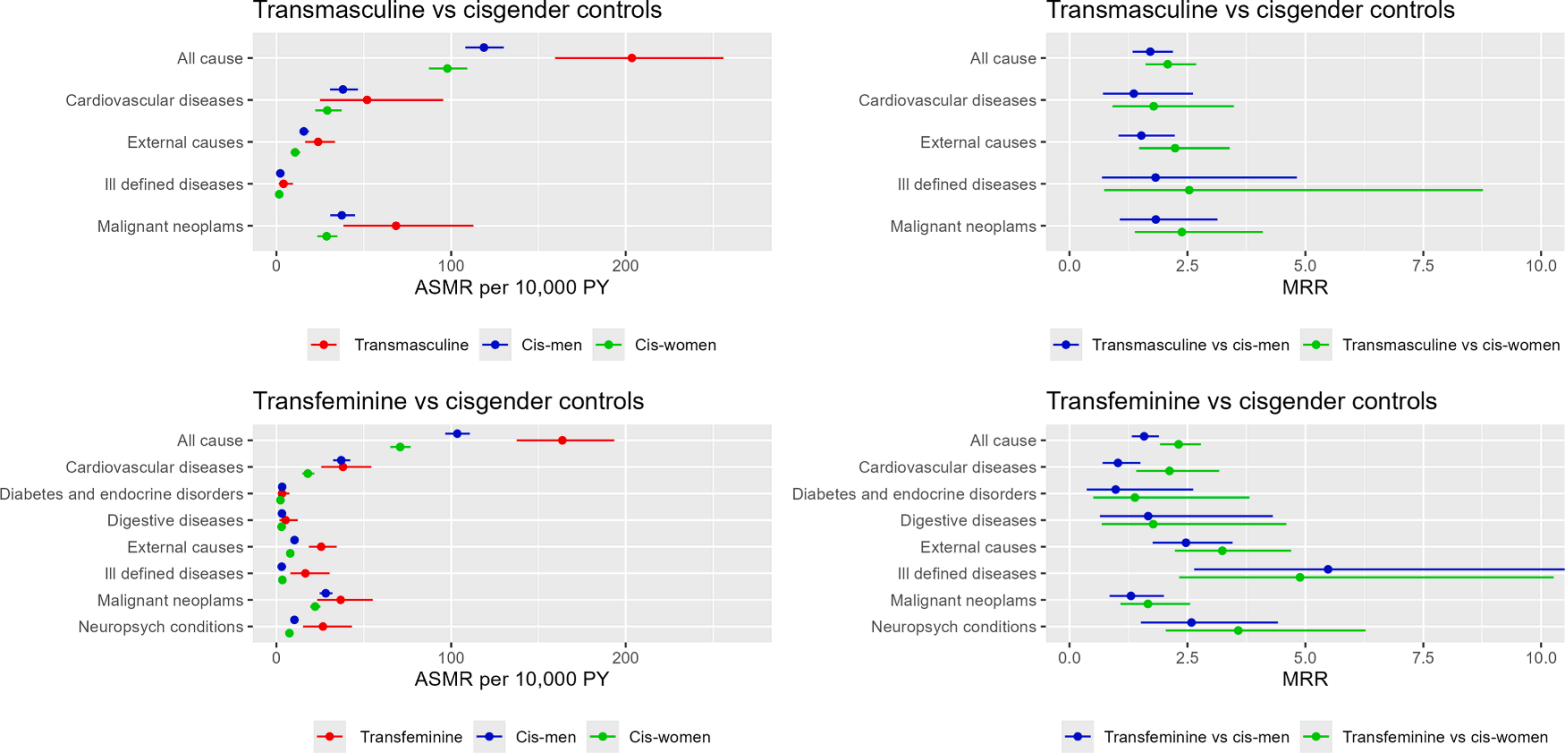
A forest plot of age-standardized mortality rates per 10,000 person-years and mortality rate ratios among all-cause and cause-specific outcomes for transgender and gender diverse people compared to cisgender people as baseline. Footnote: Outcomes included in the figures were those with ≥5 observations among transgender and gender diverse people. Blue = cisgender men, or cisgender men as baseline in MRR figure, Green = cisgender women, or cisgender women as baseline in MRR figure.

Among transfeminine people (*n* = 4181), the all-cause MRR was significantly higher compared to both cisgender women (MRR = 2.3; 95% CI 1.9, 2.8) and cisgender men (MRR = 1.6; 95% CI 1.3, 1.9; Table 2; Fig. 1). An elevated mortality risk was also observed across multiple cause-specific categories: external causes (compared to cisgender women: MRR = 3.2, 95% CI 2.2, 4.7; cisgender men: MRR = 2.5, 95% CI 1.8, 3.5), ill-defined diseases (cisgender women: MRR = 4.9, 95% CI 2.3, 10.3, cisgender men: MRR = 5.5, 95% CI 2.6, 11.4), neuropsychiatric conditions (cisgender women: MRR = 3.6, 95% CI 2.0, 6.3; cisgender men: MRR = 2.6, 95% CI 1.5, 4.4), and nutritional conditions (cisgender women: MRR = 22.0, 95% CI 2.0, 242.9; cisgender men: MRR = 27.1, 95% CI 1.7, 433.0). For cardiovascular diseases (cisgender women: MRR = 2.1, 95% CI 1.4, 3.2; cisgender men: MRR = 1.0, 95% CI 0.7, 1.5) and neoplasms (cisgender women: MRR = 1.7, 95% CI 1.1, 2.5; cisgender men: MRR = 1.3, 95% CI 0.8, 2.0), the risk for transfeminine people was higher compared to cisgender women, but there was no statistically significant relationship compared to cisgender men. The highest risk for transfeminine people's mortality based on AMRD was cardiovascular disease when compared to cisgender women (AMRD = 20.1, 95% CI 6.1, 34.1; Supplemental Table S2). However, this was closely followed by neuropsychiatric conditions, which was higher compared to both cisgender women (AMRD = 19.2, 95% CI 6.0, 32.4) and cisgender men (AMRD = 16.3, 95% CI 3.1, 29.5). Among transfeminine people, the mean YPLL difference per decedent was 8.8 years higher when compared to matched cisgender women and 6.3 years higher compared to matched cisgender men. Assuming that the observed excess years lost applies to the entire transfeminine population in Sweden, the projected total YPLL is 36,792.8 years compared to cisgender women, and 26,340 years compared to cisgender men.

**Table 2:** Overall and cause-specific age-standardized mortality rate and mortality rate ratios between transfeminine people (*n* = 4181) as compared to cisgender people (cisgender women: *n* = 41,654, cisgender men: *n* = 41,659) in Sweden from 2001 to 2023.

Cause of death	Died	Age-standardized mortality rate (per 10,000 person-years)			Mortality rate ratio (aMRR) 95% Confidence Interval	
		Transfeminine people	Cisgender women	Cisgender men	Compared to cisgender women	Compared to cisgender men
All cause	138	163.7 (137.6, 193.5)	70.8 (65.2, 76.8)	103.5 (96.6, 110.8)	**2.3 (1.9, 2.8)**	**1.6 (1.3, 1.9)**
Certain infectious and parasitic diseases	<5	4.5 (0.5, 16.2)	1.4 (0.7, 2.6)	2.1 (1.1, 3.5)	3.1 (0.7, 14.0)	2.2 (0.5, 9.5)
Cardiovascular diseases	30	38.1 (25.7, 54.4)	18.0 (14.8, 21.6)	37.1 (32.4, 42.2)	**2.1 (1.4, 3.2)**	1.0 (0.7, 1.5)
Congenital anomalies	0	0	0.1 (0.0, 0.4)	0.1 (0.0, 0.3)	0.0 (null)	0 (null)
Digestive system diseases	5	5.2 (1.7, 12.2)	2.9 (2.0, 4.3)	3.1 (2.1, 4.5)	1.8 (0.7, 4.6)	1.7 (0.6, 4.3)
Endocrine, nutritional, and metabolic diseases	5	3.2 (1.0, 7.5)	2.3 (1.3, 3.8)	3.3 (2.0, 5.1)	1.4 (0.5, 3.8)	1.0 (0.4, 2.6)
External causes	43	25.6 (18.5, 34.5)	7.9 (6.3, 9.9)	10.4 (8.8, 12.1)	**3.2 (2.2, 4.7)**	**2.5 (1.8, 3.5)**
Genitourinary diseases	0	0	0.5 (0.1, 1.4)	0.1 (0.0, 0.3)	0	0
Ill-defined diseases	10	16.6 (7.7, 30.5)	3.4 (2.1, 5.1)	3.0 (2.0, 4.4)	**4.9 (2.3, 10.3)**	**5.5 (2.6, 11.4)**
Maternal conditions	0	0	0.0 (0.0, 0.1)	0	0.0 (null)	0 (null)
Musculoskeletal diseases	0	0	0.5 (0.1, 1.2)	0.1 (0.0, 0.3)	0.0 (null)	0 (null)
Neoplasms	23	36.8 (23.3, 55.2)	22.1 (19.3, 25.2)	28.2 (24.7, 32.1)	**1.7 (1.1, 2.6)**	1.3 (0.8, 2.0)
Neuropsychiatric conditions	16	26.6 (15.2, 43.2)	7.4 (5.5, 9.8)	10.3 (8.2, 12.8)	**3.6 (2.0, 6.3)**	**2.6 (1.5, 4.4)**
Nutritional conditions	<5	4.1 (0.1, 22.6)	0.1 (0.0, 0.7)	0.2 (0.0, 0.8)	**22.0 (2.0, 242.9)**	**27.1 (1.7, 433.0)**
Respiratory system diseases	<5	3.2 (0.7, 9.2)	3.7 (2.4, 5.5)	5.7 (3.8, 8.0)	0.8 (0.3, 2.8)	0.6 (0.2, 1.8)
Skin diseases	0	0	0.2 (0.0, 1.3)	0.3 (0.0, 1.4)	0.0 (null)	0 (null)

^a^ Bolded values indicate statistical significance.

Among transmasculine people (*n* = 4857), the all-cause MRR was significantly higher compared to both cisgender women (MRR = 2.1; 95% CI 1.6, 2.7) and cisgender men (MRR = 1.7; 95% CI 1.3, 2.2; Table 3; Fig. 1). An elevated mortality risk was also observed across multiple cause-specific categories: certain infectious and parasitic diseases (compared to cisgender women: MRR = 6.0; 95% CI 1.5, 24.0; cisgender men: MRR = 15.4, 95% CI 3.5, 70.0), respiratory system diseases (cisgender women: MRR = 5.8, 95% CI 1.8, 18.4; cisgender men: MRR = 3.7, 95% CI 1.2, 11.3), external causes (cisgender women: MRR = 2.2, 95% CI 1.5, 3.4; cisgender men: MRR = 1.5, 95% CI 1.1, 2.2) and neoplasms (cisgender women: MRR = 2.4; 95% CI 1.4, 4.1; cisgender men: MRR = 1.8, 95% CI 1.1, 3.1). The highest risk for transmasculine people's mortality based on AMRD was neoplasms when compared to cisgender women (AMRD = 39.7, 95% CI 4.6, 74.8), although there was no statistically significant difference compared to cisgender men (Supplemental Table S2). Among transmasculine people, the mean YPLL difference per decedent was 9.0 years higher when compared to matched cisgender women and 2.7 years higher compared to matched cisgender men. Assuming that the observed excess years lost applies to the entire transmasculine population in Sweden, the projected total YPLL is 43,710 years compared to cisgender women, and 13,114 years compared to cisgender men.

**Table 3:** Overall and cause-specific age-standardized mortality rate and mortality rate ratios between transmasculine people (*n* = 4857) as compared to cisgender people in Sweden from 2001 to 2023 (cisgender women: *n* = 48,259, cisgender men: *n* = 48,244).

Cause of death	Died	Age-standardized mortality rate (per 10,000 person-years)			Mortality rate ratio (aMRR) 95% Confidence Interval	
		Transmasculine people	Cisgender women	Cisgender men	Compared to cisgender women	Compared to cisgender men
All causes	73	203.5 (159.6, 255.9)	97.8 (87.3, 109.3)	118.9 (108.2, 130.3)	**2.1 (1.6, 2.7)**	**1.7 (1.3, 2.2)**
Certain infectious and parasitic diseases	<5	18.9 (3.9, 55.3)	3.1 (1.0, 7.2)	1.2 (0.3, 3.2)	**6.1 (1.5, 25.7)**	**15.4 (3.5,70.0)**
Cardiovascular diseases	10	51.9 (24.9, 55.3)	29.1 (22.3, 37.4)	38.1 (30.7, 46.7)	1.8 (0.9, 3.5)	1.4 (0.7, 2.6)
Congenital anomalies	0	0	0.0 (0.0, 0.7)	0.0 (0.0, 0.1)	0.0 (null)	0.0 (null)
Digestive system diseases	<5	0.6 (0.0, 3.4)	2.4 (1.1, 4.5)	2.2 (1.2, 3.8)	0.3 (0.0, 2.0)	0.3 (0.0, 2.1)
Endocrine, nutritional, and metabolic diseases	<5	13.6 (6.4, 33.5)	2.0 (0.8, 4.2)	2.7 (1.2, 5.0)	6.7 (0.8, 54.3)	5.1 (0.6, 40.5)
External causes	33	23.9 (16.4, 33.5)	10.7 (8.3, 13.6)	15.7 (13.1, 18.6)	**2.2 (1.5, 3.4)**	**1.5 (1.1, 2.2)**
Genitourinary diseases	0	0	1.1 (0.1, 4.2)	1.5 (0.2, 5.3)	0	0
Ill-defined diseases	5	4.1 (1.3, 9.5)	1.6 (0.5, 3.7)	2.2 (1.4, 3.4)	2.5 (0.7, 8.8)	1.8 (0.7, 4.8)
Maternal conditions	0	0	0.0 (0.0, 0.1)	0	0.0 (null)	0.0 (null)
Musculoskeletal diseases	0	0	0.5 (0.0, 2.9)	0	0.0 (null)	0.0 (null)
Neoplasms	15	68.4 (38.3, 112.9)	28.7 (23.4, 34.9)	37.4 (30.8, 45.0)	**2.4 (1.4, 4.1)**	**1.8 (1.1, 3.1)**
Neuropsychiatric conditions	<5	0.1 (0.0, 0.8)	14.2 (10.1, 19.4)	11.9 (8.9, 15.5)	<0.1 (0.0, 0.1)	<0.1 (0.0, 0.1)
Nutritional conditions	0	0	0	0.0 (0.0, 0.1)	0	0
Respiratory system diseases	<5	22.0 (6.0, 56.3)	3.8 (1.8, 7.0)	6.0 (3.3, 10.0)	**5.7 (1.8, 18.3)**	**3.7 (1.2, 11.1)**
Skin diseases	0	0	0.5 (0.0, 2.9)	0	0.0 (null)	0.0 (null)

^a^ Bolded values indicate statistical significance.

## Discussion

Mortality was higher among TGD people for many causes of death compared to both matched cisgender men and cisgender women. Among transfeminine people, the risks of mortality attributed to external causes, ill-defined diagnoses, and neuropsychiatric conditions were elevated compared to both cisgender comparison groups, while the risks of death due to cardiovascular diseases and neoplasms were only elevated compared to cisgender women. Among transmasculine people, the risks of mortality were elevated for certain infectious and parasitic diseases, neoplasms, and respiratory system diseases compared to both cisgender comparison groups. However, mortality risks from infectious and parasitic diseases, respiratory system diseases, and nutritional conditions should be interpreted with caution due to the very low number of cases.

Compared to prior research in Sweden, which included only people from 1973 to 2003 who had legally changed gender and a limited number of outcomes,^6^ this study represents a broader, more timely cohort. All-cause MRR and some cause-specific MRR were elevated in both studies, although the difference between TGD people and cisgender people was smaller in the present study compared to the previous Swedish study.^6^ Yet this should be interpreted with caution, as the present study applied age-standardization. Unlike a recent UK study (data from 1988 to 2019) that adjusted for a wide range of confounders such as race/ethnicity, socioeconomic status, and health behaviors,^5^ our study was limited to matching on age and county due to data availability and reliability in the Swedish population registers. This may contribute to the higher all-cause MRR for both transfeminine and transmasculine people observed in the present study in comparison to the UK study. A Dutch study using data from 1972 to 2018 and an age-standardized approach observed similar all-cause MRR^10^; however, this study sample was limited to solely TGD people receiving hormonal treatments, whereas the present study examined the total population of people with a gender dysphoria diagnosis in Sweden. MRRs in the Dutch study were also elevated for external causes among TGD people compared to at least one cisgender group; whereas this was elevated in the present study compared to all cisgender comparison groups. Ill-defined diseases were not an outcome included in the previous Swedish or Dutch studies and, subsequently, cannot be compared.^6^^,^^10^ An increased MRR due to ill-defined diseases for both transmasculine and transfeminine people was also observed in the UK study, although only when compared to cisgender men. In the present study, this difference was only observed for transfeminine people, but was elevated compared to both cisgender women and cisgender men.^5^

Our finding that transfeminine people, but not transmasculine people, have a higher risk of mortality due to cardiovascular diseases was also reported by a Dutch study that examined mortality among TGD people receiving gender affirming hormone therapy (GAHT),^10^ but not in a UK study.^5^ This increased risk may be due to increased prevalence of cardiovascular disease, often presumed to be due to GAHT, though the evidence is mixed. Some studies have found an association between GAHT containing ethinyloestradiol and cardiovascular disease risk,^30^^,^^31^ while others have not.^11^^,^^12^ In recent years, lower dose transdermal or oral bioidentical estrogen has replaced ethinyloestradiol and is part of the praxis in Sweden. This is not associated with an increase cardiovascular disease prevalence among transgender women receiving GAHT compared to transgender women not receiving GAHT.^31^^,^^32^ However, data from a US population-based survey reported that transfeminine people, regardless of GAHT use, had an increased prevalence of heart disease, stroke, and myocardial infarction compared with cisgender women and an increased prevalence of any cardiovascular disease compared with cisgender men.^33^^,^^34^ A meta-analysis reported that the overall incidence of cardiovascular disease was greater in transgender women than cisgender men, driven largely by an increased risk of thrombosis^31^; however, the present study showed no difference in cardiovascular risk when compared to cisgender men. This could be due to changes in prescribing norms or other improvements in risk factors not measured in the present study.

In the present analysis, risk of death from neoplasms was elevated in transmasculine people compared to both cisgender women and men, and among transfeminine people compared to cisgender women. These findings contrast with previous mortality research, which found a reduced or null risk of cancer deaths for all TGD people^11^^,^^12^ or at least transmasculine people.^5^^,^^10^ However, similar to our findings, previous Swedish and Dutch studies found an increased risk of cancer mortality for transfeminine people.^6^^,^^10^ Smoking prevalence has been reported to be higher among TGD people than cisgender people.^35^, ^36^, ^37^ One US study found that TGD people may be more likely to be diagnosed with cancer at advanced stages,^38^ contributing to increased mortality in this population. Disparities in cancer staging at time of diagnosis should be examined in future research in Sweden to determine if this is a contributing factor. The observation that transfeminine people were not at increased risk of neoplasm mortality compared to cisgender men, may be due to a lower incidence of prostate cancer (the second leading cause of cancer death among cisgender men) because of antiandrogen and estrogen hormone therapy use in this population as part of GAHT that has been shown to have a protective effect on this type of cancer specifically.^39^

An elevated risk of external causes of death (which includes suicide, homicide, and accidental poisonings) has also been noted across many mortality studies. Data from the US Veterans Affairs report nearly 3-fold increased risk of suicide among all TGD persons,^8^ while a previous Swedish study found a 19-fold increased risk.^6^ A Danish registry study from 1980 to 2021 found suicide mortality was 3.5 times higher among TGD people compared to cisgender people.^7^ They also found that the risk of a previous suicide attempt was 7.7 times higher among TGD people than cisgender people. A Dutch study of TGD people who have received gender-affirming treatments between 1972 and 2018 disaggregated by sex assigned at birth and found a 3- to 7-fold increased risk of suicide among transgender women, but no significant increase among transgender men.^10^ In England, the risk of death from external causes was elevated among both groups of TGD people, but only when compared to cisgender women.^5^ In the present study, both transfeminine people and transmasculine people were at greater risk of mortality due to external factors such as suicide when compared to both cisgender men and women.^40^ A European Union (EU) survey on experiences in the sexual minority and TGD communities found that TGD respondents reported high levels of discrimination, harassment, and violence that have increased over recent years.^41^ Such exposure to minority stress, or excess stress related to social marginalization, is associated with increased suicide risk and likely contributes to both increased CVD and cancer mortality risk^42^; yet, measurement of these factors is not presently possible within register data.

The present study observed divergent patterns in mortality outcomes between transfeminine and transmasculine people. Transfeminine people had increased risk for some outcomes when compared to cisgender women but not cisgender men, such as cardiovascular diseases and neoplasms. Yet, transmasculine people consistently had a higher risk for mortality outcomes when compared to both cisgender men and cisgender women control groups. This divergence underscores the limitation of pooling transgender cases or cisgender controls, which would obscure these nuanced, outcome-specific trajectories. Consequently, these findings serve as support for future research to investigate the specific biological, behavioral, or social mechanisms driving these unique risk patterns, rather than assuming a uniform effect of gender identity across all causes of death.

To our knowledge, this study provides the most current and comprehensive registry-based national estimates of all-cause and cause-specific mortality among TGD people in Sweden who have had contact with the gender-affirming healthcare system. A key strength is that the study uses a large, population-based sample and age and county-matched cisgender people as control groups. This facilitated robust comparisons between populations and across sex assigned at birth. The inclusion of ASMR and YPLL provides a nuanced depiction of mortality risk for TGD individuals. The total population YPLL projection quantifies the potential loss if current mortality patterns persist, estimating the burden on the broader community in relation to cisgender people of similar age and county of residence. However, the study is limited by the lack of data on potential confounders, i.e., smoking and alcohol use, which may influence the study's findings. Additionally, using diagnostic codes to identify TGD persons omits people who have not accessed formal care or who are misclassified in the registers. This may impact the representation of mortality risk due to external causes, as people awaiting evaluation for a gender dysphoria diagnosis would not be included and may be vulnerable to this outcome.^43^, ^44^, ^45^ The TGD people included in the present study are young, with a mean age of 25 years, which likely underestimates mortality from age-related causes that typically increase later in life. While the risk of bias is reduced due to the matching of cisgender people by age and county of residence, there is still a need to continue to monitor mortality outcomes as this cohort ages. Further, it is not possible to determine if there were differences in mortality outcomes following the end of sterilization requirements of legal gender recognition in 2013 due to the relatively young age of the cohort and limited time that has passed. Importantly, while this study identifies significant mortality disparities, the observational nature of registry data limits our ability to establish causality. The elevated risks observed reflect the complex relationships between biological, behavioral, and social factors that require further investigation.

The results of this study provide important opportunities in the pursuit of health equity for TGD people. The increased MRR related to ill-defined diseases observed among transfeminine people requires additional exploration. Deaths that occur and require a medical examiner's involvement may be given an ICD-10 code in this category. This code should be changed following completion of the examination as required by Swedish law,^25^ but delays in completion, mismatches with personal identification numbers following legal gender changes, or other bureaucratic insufficiencies could contribute to this variation. Therefore, these may be deaths due to external causes not classified, or updated, as such. However, one would anticipate, then, that these differences would also be observed among transmasculine people. A recent study examining legal gender changes showed no difference in legal gender change utilization based on sex assigned at birth.^46^ This may be an indication of systemic barriers or stigma during post-mortem investigations,^3^^,^^22^^,^^23^ although this requires further study; therefore, disparities observed in deaths due to ill-defined diseases should be interpreted with caution. Differences observed in certain infectious or parasitic diseases among transmasculine people could prompt further exploration. Recent studies have pointed to decreased immune response among transmasculine people receiving testosterone therapy, bringing risk for such outcomes closer to that of cisgender men,^47^ possibly explaining the present findings. Although it is important to note that the confidence interval was notably wide due to so few cases.

Unlike previous studies limited to people receiving hormonal treatments or who have legally changed gender, this study captures a broader and more representative cohort of TGD people in Sweden. Including both absolute and relative measures of mortality and stratification by sex assigned at birth, offers a more nuanced understanding of mortality disparities. The analyses showed elevated risks across nearly all major mortality outcomes compared to cisgender controls as well as differences by sex assigned at birth, with transfeminine and transmasculine people experiencing distinct mortality patterns. These findings underscore the persistence of health disparities, particularly in the mortality risk due to cardiovascular diseases, certain infectious diseases, external causes, ill-defined conditions, neoplasms, and neuropsychiatric diseases. The increased prevalence of neuropsychiatric conditions may be misclassified as external causes, as the causes of death included in this category include alcohol use disorders and bipolar and depressive disorders; people with these conditions have a higher incidence of suicide and overdose deaths than those without.^24^^,^^48^^,^^49^ The predominance of suicide and the high proportion of deaths coded as ill-defined conditions suggest critical gaps in mental health support and possibly postmortem investigation practices. These results quantified the burden of premature mortality but do not identify the specific mechanisms driving these disparities. While targeted public health interventions are needed, our registry data alone cannot distinguish whether these excess deaths result from biological factors, behavioral risks, systemic barriers, or administrative biases. Future research linking clinical treatment records to mortality data is required to disentangle the roles of medical interventions, social determinants, and healthcare access. Thus, the present findings serve primarily as a baseline for monitoring mortality trends rather than evidence of specific causal pathways. Cumulatively, these findings contribute to a growing international body of evidence on TGD health disparities and align with global calls for inclusive and equitable healthcare systems.

## Contributors

FCP is the senior author. KDC, RW, and FCP designed the study. KDC took the lead in drafting the manuscript. KDC and RW conducted the statistical analysis, interpreted the results, and verified the underlying data. SSJ, AS, and TF provided substantial input on the scientific approach, interpretation of study results, and revision of the manuscript. KDC and FCP are the guarantors. The corresponding author attests that all listed authors meet authorship criteria and that others who meet authorship criteria have not been omitted. All authors read and approved the final version of the manuscript.

## Data sharing statement

Our study includes data from Swedish National Registers, which cannot be directly shared due to confidentiality concerns and agreements. Data can be requested from the National Board of Health and Welfare in Sweden (registerservice@socialstyrelsen.se) and Statistics Sweden (https://www.scb.se/om-scb/kontakta-oss/statistikservice/fraga-oss).

## Declaration of interests

All authors have completed the Competing Interest form (available on request from the corresponding author) and declare: Support from the Forte Research Council was received for the present project, AS reports payment for lectures from SFOG (Swedish Society of Obstetrics and Gynecology), less than USD 1500 per year. All other authors declare no competing interests.
