# Supplementary material for: Nationwide estimates of all-cause and cause-specific mortality among individuals with gender dysphoria in Sweden, 2001–2023: a register-based cohort study
Source: eClinicalMedicine. 2026 Jul 31;98:104088. doi: 10.1016/j.eclinm.2026.104088 (PMC13453480; doi:10.1016/j.eclinm.2026.104088)
Supplement: Supplementary file 1 [file mmc1.docx]

Supplemental Table 1: ICD codes and mortality outcomes

| Mortality Outcome | ICD-10 Code |
| --- | --- |
| Certain infectious and parasitic diseases | A00-B99, G00-G04, G14, N70-N73, P37.3, P37.4 |
| Cardiovascular diseases | I00-I99 |
| Congenital anomalies | Q00-Q99 |
| Diabetes mellitus and endocrine disorders | E10-E14, D55-D64 (without D64.9), D65-D89, E03-E07, E15-E16, E20-E34, E65-E88 |
| Digestive system diseases | K20-K93 |
| External causes | V, W, X00-X40, X43, X46-Z99, Y00-Y89, Y870-Y872 |
| Genitourinary diseases | N00-N64, N75-N98 |
| Ill-defined diseases | R00-R94, R96-R99 |
| Maternal conditions | O00-O99 |
| Musculoskeletal diseases | M00-M99 |
| Neoplasms | C00-C97, D00-D48 |
| Neuropsychiatric conditions | F01-F99, G06-G98 (minus G14), U07.0, X41, X42, X44, X45 |
| Nutritional Deficiencies | D50-D53, E00-E02, E40-E46, E50, E51-E64 |
| Oral conditions | K00-K14 |
| Perinatal conditions | P00-P96 (minus P23, P37.3, P37.4) |
| Respiratory system diseases | J30-J98 |
| Sense organ diseases | H00-H61, H68-H93 |
| Skin diseases | L00-L98 |

Supplemental Table 2. Overall and cause-specific absolute mortality rate differences between transfeminine individuals (*n* = 4,181) as compared to cisgender individuals (cisgender women: *n* = 41,654, cisgender men: *n* = 41,659) and transmasculine individuals (*n* = 4,857) as compared to cisgender individuals (cisgender women: *n* = 48,259, cisgender men: *n* = 48,244) in Sweden from 2001-2023.

| **Cause of Death** | **Died** | **Absolute Mortality Rate Difference**  **(per 10,000 person-years)** | | **Absolute Mortality Rate Difference**  **(per 10,000 person-years)** | |
| --- | --- | --- | --- | --- | --- |
|  |  | **Transfeminine Individuals** | | **Transmasculine Individuals** | |
|  |  | **Compared to cisgender women** | **Compared to cisgender men** | **Compared to cisgender women** | **Compared to cisgender men** |
| All causes | 73 | **92.9 (65.0, 120.8)** | **60.2 (32.0, 88.4)** | **105.7 (57.8, 153.7)** | **84.7 (36.7, 132.6)** |
| Certain infectious and parasitic diseases | <5 | 3.0 (-3.2, 9.3) | 2.4 (-3.9, 8.7) | 15.8 (-5.7, 37.4) | 17.7 (-3.8, 39.1) |
| Cardiovascular diseases | 10 | **20.1 (6.1, 34.1)** | 1.0 (-13.4, 15.4) | 22.8 (-10.2, 55.8) | 13.8 (-19.3, 46.9) |
| Congenital anomalies | 0 | -0.1 (null) | -0.1 (null) | 0.0 (null) | 0.0 (null) |
| Digestive system diseases | <5 | 2.3 (-2.4, 7.0) | 2.1 (-2.6, 6.8) | -1.8 (-3.7, 0.2) | -1.6 (-3.3, 0.0) |
| Endocrine, nutritional, and metabolic diseases | <5 | 0.9 (-2.1, 3.9) | -0.1 (-3.2, 3.1) | 11.6 (-15.1, 38.2) | 10.9 (-15.8, 37.6) |
| External causes | 33 | **17.7 (9.9, 25.6)** | **15.2 (7.4, 23.1)** | **13.2 (4.7, 21.8)** | 8.2 (-0.4, 16.8) |
| Genitourinary diseases | 0 | -0.5 (null) | -0.1 (null) | -1.1 (null) | -1.4 (null) |
| Ill-defined diseases | 5 | **13.2 (2.8, 23.5)** | **13.5 (2.2, 23.9)** | 2.5 (-1.4, 6.3) | 1.8 (-1.8, 5.5) |
| Maternal conditions | 0 | 0.0 (null) | 0 (null) | 0.0 (null) | 0.0 (null) |
| Musculoskeletal diseases | 0 | -0.5 (null) | -0.1 (null) | -0.5 (null) | 0.0 (null) |
| Neoplasms | 15 | 14.7 (-0.6, 30.0) | 8.5 (-6.9, 24.0) | **39.7 (4.6, 74.8)** | 31.0 (-4.3, 66.4) |
| Neuropsychiatric conditions | <5 | **19.2 (6.0, 32.4)** | **16.3 (3.1, 29.5)** | **-14.0 (-18.5, -9.6)** | **-11.7 (-14.9, -8.5)** |
| Nutritional conditions | 0 | 3.9 (-4.1, 11.8) | 3.9 (-4.0, 11.9) | 0.0 (null) | 0.0 (null) |
| Respiratory system diseases | <5 | -0.6 (-4.4, 3.3) | -2.5 (-6.6, 1.6) | 18.2 (-3.5, 39.8) | 16.0 (-5.8, 37.8) |
| Skin diseases | 0 | -0.2 (null) | -0.1 (null) | 0.0 (null) | 0.0 (null) |
